# Peritoneal Dialysis Drop-out: Causes and Prevention Strategies

**DOI:** 10.4061/2011/434608

**Published:** 2011-10-27

**Authors:** Kunal Chaudhary

**Affiliations:** ^1^Division of Nephrology, Harry S. Truman Veterans Medical Center, Columbia, MO 65203, USA; ^2^Division of Nephrology, Department of Internal Medicine, University of Missouri, 1 Hospital Drive, CE 422, Columbia, MO 65212, USA

## Abstract

Peritoneal dialysis (PD) as a renal replacement therapy (RRT) has become wide spread since its inception more than twenty-five years back. Since then, several advances have been made and PD has been accepted as an alternative therapy to hemodialysis (HD), with excellent survival, lower cost, and improved quality of life. In spite of comparable survival of HD and PD, improved PD techniques over the last few years, and lower health care costs with PD, PD prevalence remains low in many countries. An important reason for the low PD prevalence is patient dropouts, that is, transfer to HD. The reasons for dropouts are multifactorial, that is, modality related, system related, and patient related. These include episodes of peritonitis, catheter-related problems, ultrafiltration failure, patient fatigue, and provider comfort. This review discusses the various factors that contribute to PD dropout and the strategies to prevent it.

## 1. Introduction

Peritoneal dialysis (PD) has been in use for the last thirty years for treatment of end-stage renal disease but barring a few countries the prevalence of PD is lower than Hemodialysis (HD). For example as per USRDS data from 2007, of approximately 368,000 patients undergoing dialysis in the United States (USA), the point prevalence for PD patients was only 7.2% [1]. Contrary to prevailing practice, a survey of nephrology professionals found that the majority picked CAPD/APD as the best initial therapy for the patient [2]. Of the ones who perform PD, few patients stay on PD for 5 years or longer from initiation of therapy. A large proportion of such patients transfer from PD to HD every year, and PD to HD switch rates of more than 35% have been reported [3, 4]. Thus transfer to HD is a significant cause for the low prevalence of PD. The risk of transfer to HD is the highest in the first few months of PD initiation, mainly due to episodes of peritonitis and catheter-related problems and decreases thereafter [5]. Modality issues such as recurrent episodes of peritonitis, inadequate dialysis or ultrafiltration failure, system issues such as lack of infrastructure as well as personal or social reasons make up the bulk of causes for transfer to HD (Table 1). Peritonitis and inadequate dialysis issues have received great attention and despite the incidences having decreased the last few years, PD drop-out still remains widespread. Strategies to prevent and manage peritonitis, ultrafiltration failure, catheter-related complications, and improving adequacy of dialysis, education of patients, and medical staff may all help with maintaining the patient on PD (Table 2).

## 2. Reasons for PD Drop-out

### 2.1. Modality-Related Issues

#### 2.1.1. Peritonitis and Catheter-Related Infections

A major cause for the transfer from PD to HD is high rates of peritonitis, especially within the first 1 to 2 years of initiating PD. Peritonitis episodes, even if not the approximate cause of technique failure (TF), can cause ultrafiltration failure (UF) and membrane-related problems at a later time. The use of twin bags and Y set system has helped decrease the peritonitis rates significantly in the last few years [6]. Data from Canadian Organ Replacement Registry (CORR) from 1981 to 1997 estimated the crude CAPD switch rates to be 154/1000 patient-year. Compared to earlier years, the adjusted relative risk of CAPD failure ranged from 0.75 to 0.83 for the years 1990 and onwards [7]. In another prospective study of 292 PD patients involving 28 dialysis centers, 24.8% PD patients switched to HD during the study period and 40% of those patients switched within the first year and 70% within two years of starting PD [8]. In their series, the most common cause for the switch was infection related (both peritonitis and catheter related) at 36.9% followed by volume overload at 18.5%. Peritonitis rates in randomized trials using double bag systems have shown an incidence rate of peritonitis ranging from one episode per 24.8 months to one episode per 46.4 months [9]. In a study of peritonitis rates in 12 PD units in the United Kingdom (UK), the author reported peritonitis rates of one episode per 14.7 months for CAPD and one episode per 18.1 month for APD/CCPD patients, with a considerable variation between units [10]. Catheter-related interventions, including changes in design and approach have been tried in an effort to reduce peritonitis rates. In a review of 37 trials including 2822 patients Strippoli et al. did not find statistical difference in peritonitis rates, or technique failure with catheters inserted by laparoscopy versus laparotomy or between catheters with a straight versus coiled intraperitoneal portion [6]. In a comparison of pre-sternal versus intraperitoneal PD catheters, Twardowski et al. found that the peritonitis rate was 1 episode per 37.4 patient-months and 1 per 20.5 patient-months for presternal and abdominal catheters, respectively, but these differences were not statistically significant. However, the patients receiving the presternal catheters included obese patients (5 patients with BMI > 45), and 3 patients had ostomies [11].

#### 2.1.2. Ultrafiltration Failure and Volume Overload

Inability to maintain adequate volume status is another cause of failure of PD as a modality. This could be due to several reasons including ultrafiltration failure, decline in RRF, or excessive salt and water intake. The diagnosis of true ultrafiltration failure as per ISPD guidelines can be ascertained using 4.25% dextrose solution for 4 hours and getting >400 mL of net ultrafiltration [12]. The prevalence of ultrafiltration failure as a cause of TF has been reported to be between 1.7% and 13.7% [13]. A study of PD cohort followed in the Netherlands had a high rate of technique failure, with only 64% of patients remaining on PD after 2 years [14]. The factors identified as independent predictors for this were urine volume, systolic blood pressure, and peritoneal ultrafiltration. In a study from Japan, the authors reported that failure of ultrafiltration was the biggest reason for withdrawal from CAPD in patients staying on PD for greater than 6 years [15]. In both these studies the major modality was CAPD and Icodextrin was not used. However, recent studies have shown that cohorts of incident PD patients starting dialysis from 2002 onwards had lower rates of transfer to PD as compared to cohorts from earlier years [16, 17]. Long-term exposure to hypertonic glucose solutions changes the transport characteristics of peritoneal membrane. Low or average transporters become high transporters and may lead to a greater use of high-strength dextrose solutions. The resulting volume expansion is often compounded over time as the residual renal function (RRF) declines over time as well. Volume overload may be due to causes besides peritoneal membrane dysfunction. Dietary indiscretion, excessive sodium, and fluid intake; an inadequate dialysis prescription; loss of residual renal function without adjustments in dialysis prescription; catheter malfunction often are the cause of inadequate volume control.

#### 2.1.3. Catheter Malfunction

Mechanical complications of PD catheters are another reason for failure of PD and transfer to HD. Migration of catheters and blockage due to omental trapping are frequent causes of malfunction. Early and appropriate intervention can save many catheters, often without interrupting PD [18].

### 2.2. System-Related Issues

Compared to some countries such as Canada or UK, where accessibility to HD can be limited, HD is more readily available in the USA [19]. Thus, prevalence of PD is higher (20%–30%) in Australia, New Zealand, China, Canada, and the UK, where PD delivery is supported and provided for by the government [20, 21]. In Hong Kong, where 80% of the dialysis population is on PD (HD is only permitted if there is a contraindication to PD) a 2-year technique survival of 82% (patient survival 91%) has been reported [22]. Besides reimbursement policies and possible genetic effects [23] the success of PD in Hong Kong is due to the high numbers of PD patients each unit has, that is, around 300, increasing the staff expertise. For example the training duration for PD is only 4-5 days, and many procedures such as catheter insertion and removal are performed by nephrologists, decreasing surgical consultations, and providing timely treatment.

In a recent study, during the 9-year period between 1996 and 2004, the number of units owned by large dialysis organizations (LDOs) in the USA increased by >50% [24]. The number of patients undergoing dialysis in these units increased from 39% to 63% with no increase in number of patients undergoing PD. Three of the five LDOs in the above study had consistently lower peritoneal dialysis patients and higher risk of death in those patients than the other LDO and non-LDO owned units. It has been reported that the drop-out rates are higher in centers having fewer number of PD patients, generally <20–25 [3, 4, 25]. Studies have linked the low number of peritoneal dialysis patients in a center to high technique failure and low patient survival [26, 27]. Thus a vicious cycle can develop where low numbers of peritoneal dialysis patients lead to lack of training/expertise [25–28], which in turn affects the ability to problem solve in the face of technique failure leading to patient drop-out. In a Program Director survey, 29% of US training programs had less than five chronic peritoneal dialysis patients per nephrology trainee. Similarly, in 14% of US training programs, fellows spent less than 5% of their time receiving training for patients undergoing chronic peritoneal dialysis and only 32% of renal fellows stated that they attended outpatient PD clinic [29]. Inadequate training in the modality may lead to a lack of comfort with the therapy and nephrologists who are not comfortable with peritoneal dialysis might have a tendency of transferring patients to HD more readily.

### 2.3. Patient-Related Issues

Of the several patient-related factors which contribute to underutilization of PD, getting inadequate predialysis education is one. There is a strong relationship between the probabilities of offering peritoneal dialysis as a treatment option to the selection of chronic peritoneal dialysis as a treatment modality [30]. Geography and distance play an important role, and the distance to travel to the dialysis unit may be a factor [31].

In a study of the Dutch registry from 1994 to 1999, the investigators did not find diabetes and patient's sex related to technique failure but advancing age was related to TF [25]. In another study, the technique failure rates were the same with patients above and below 55 years of age. Diabetics had a slightly higher technique failure rate, but the results did not reach statistical significance [4]. In a prospective study of 262 patients, Jaar et al. [8] found that 18.2% of patients switched due to fluid overload problems. Abdominal surgeries and malnutrition were other leading causes of PD failure in the study. Although dialysis provides life-sustaining therapy for patients with irreversible renal failure, it does not restore a normal quality of life. Over time a certain level of fatigue may occur in PD patients as a result of both their disease and of their constant requirement to perform life-sustaining dialysis. This chronic patient burnout is another reason for PD drop-out especially if adequate psychosocial support is not available.

## 3. Prevention Strategies

### 3.1. Dialysis Related

#### 3.1.1. Peritonitis

Prevention and better treatment of peritonitis and catheter-related infections will undoubtedly decrease the loss of some patients from PD. Prophylaxis against exit site infection leads to subsequent fewer episodes of peritonitis and both mupirocin and gentamycin are used for this. Nasal carriage of Staphylococcus aureus has been linked to increased rates of peritonitis [32]; screening and prophylaxis with antibiotics has shown to decrease the rates of peritonitis [33, 34]. Bernardini et al. [35] shows that the use of gentamycin was more effective than mupirocin in preventing gram-negative infections and equally effective as mupirocin against gram positive organisms. In certain centers APD has been associated with lower peritonitis and technique failure [36], and more widespread use of APD can offset some of the patients failing PD. In the study discussed above [10] patients receiving both cephalosporins and a second antibiotic (i.e., gentamycin) by intraperitoneal route had the best cure rates of 94.55%, compared to patients receiving either intraperitoneal vancomycin or oral cephalosporins, and dual initial coverage until the cultures and sensitivity come back should be considered as the standard practice. Investigating the cause for variability of peritonitis rates from one center to another in a geographical region as well as reviewing training techniques and center-specific protocols periodically can help minimizing peritonitis episodes. Reduction in peritonitis rates using biocompatible solutions has not been shown in prospective randomized controlled trials (RCT). One retrospective study using biocompatible solutions showed lower rates of peritonitis rates (1 episode/36 versus 1 episode/21 patient-months), as compared to use of standard solution [37]. Another single center study of 121 cases of peritonitis also found a lower rate of peritonitis (1 episode/52.5 versus 1 episode/26.9 patient-months) in patients treated with biocompatible solutions as compared to standard solution [38]. However other studies including the Euro-balance study using biocompatible solutions have not shown any difference in peritonitis rates [39, 40]. Currently, the ISPD does not recommend use of biocompatible solutions as a measure for reducing peritonitis rates.

#### 3.1.2. Ultrafiltration Failure/Volume Status

Glucose, the osmotic agent in standard PD solutions, causes changes in the membrane over time which eventually leads to membrane failure [41]. Newer biocompatible solutions without dextrose have shown evidence of less membrane damage and might lead to better preservation of the peritoneal membrane [42]. In a Japanese cohort of greater than 7000 patients, the drop-out rate of patients who used Icodextrin (8.9%), was significantly lower than those using dextrose (14.5%), (*P* < 0.0001) [43]. In a double blind randomized trial in PD patients use of Icodextrin in long dwell versus standard 2.25% dextrose solution achieved greater ultrafiltration and sodium losses [44]. As the transport status of the patient changes and low transporters become high transporters, use of cycler can maintain the ultrafiltration and continue the patient on PD. Maintenance of RRF is of importance in PD patients, and rates of decline of RRF have been associated with all-cause mortality as well as TF [45]. The effects of newer biocompatible solutions have been studied in clinical trials such as Euro-balance which showed a significant improvement in effluent markers of peritoneal membrane integrity and significantly decreased circulating AGE levels, along with better preservation of RRF but decreased peritoneal ultrafiltration [40]. In a 12-month randomized study [39], no difference was seen for ultrafiltration volumes, urine output, and RRF in patients that used neutral solution *versus *standard solution. Another randomized controlled study of 93 incident PD patients comparing standard to biocompatible solutions did not find a difference in RRF at 3 and 12 months [46]. The use of biocompatible solutions with lower levels of glucose degradation products (GDPs) may preserve the RRF longer, although this effect may be volume related [47]. Often the PD patient's prescription does not get changed to compensate for the loss in RRF. Close attention to the prescription and to protecting the peritoneal membrane as well as patient education about diet and maintenance of dry weight and use of loop diuretic are essential in achieving normovolemia in PD patients. To maintain RRF, nephrotoxic agents such as contrast and aminoglycoside (AG) should be avoided as far as possible and used only as a short course with drug level monitoring. The dialysis prescription should be optimized to avoid dehydration and hypotension which can adversely affect the RRF. Since HD has been associated with an increased rate of loss of RRF as compared to PD [48, 49], temporary HD at dialysis initiation which may reduce the RRF should be avoided if possible [50]. The renoprotective effects of inhibitors of renin angiotensin system (RAS) may also apply to dialysis patients in preserving the RRF and improve outcomes. Two RCTs using ramipril and valsartan, respectively, have shown that in select populations their use was associated with preservation of RRF, but patients in whom these drugs could not be withdrawn were excluded from the study [51, 52]. In another study of incident PD patients, where there was no exclusion based on cardiovascular status, Kolesnyk et al. showed no decline in RRF over a 3-year period [53]. The same investigators showed that use of RAS inhibitor led to less increase in small solute transport as compared to controls [54], which may have a positive impact on PD survival. Newer solutions using a combination of crystalloid and colloid as well as low sodium solutions are being investigated in an effort to improve fluid and sodium removal [55, 56].

#### 3.1.3. Catheter Issues

Many causes of catheter malfunction, such as occlusion by bladder or bowels, can be corrected with the use of laxatives or emptying the bladder. Clots can be dislodged by injecting heparinized saline and if unsuccessful, by instillation of tPA or urokinase in the catheter. Common mechanical problems of omental trapping, adhesion formation, and so forth, can be corrected through laparoscopic means by performing omentopexy, adhesiolysis, resection of epiploic appendices, colopexy, and so forth, [57]. Radiological imaging should be done early and judiciously to get a better idea of the underlying problem, such as migrated catheters, which can also be successfully corrected by laparoscopic approach. The use of presternal catheters can allow certain type of patients, that is, obese, or ones having a colostomy an opportunity to do PD.

### 3.2. System Related

#### 3.2.1. Optimizing the PD Facility

Successful PD programs are usually of a size that allows nurses to assume primary responsibility for patient care. Optimal ratios of nurses to patients are generally felt to be about 1 : 20. Nurses develop a rapport with patients that is professionally satisfying and they have a sense of autonomy that is particularly rewarding. Thus, conceptually, programs of 50 or more PD patients would seem to be optimum. Larger programs provide flexibility with nursing on-call schedules and allows nurses time to actively participate in educational programs. The goal of a smaller program should be to try and reach this threshold and/or consider consolidating on a regional basis to provide more effective patient care. In certain parts of the world, identifying a center of excellence which can establish satellite programs has been used. One such model used in China resulted not only in rapid growth of PD but also had excellent technique survival of 93% at one year and peritonitis rates of 0.26 episodes per year at risk [58].

#### 3.2.2. Patient Education and Training

A predialysis program where patients can be referred a few months before the need for dialysis can go a long way towards preparing the patient for PD and educate them regarding possible complications [59]. Such a program should include nephrologists, nurses, dieticians, social workers, and even other dialysis patients. In a report from Hong Kong 50% of patients who were offered PD were reluctant to start PD, but agreed after predialysis counseling [60]. Another report from the United kingdom showed that close to 50% of patients who receive an explanation for both PD and HD through predialysis counseling chose PD [61]. In USA the National Pre-ESRD Education Initiative, which involved 932 referring nephrologists and 28 educators from all over the United States, is the largest pre-ESRD program undertaken to date [62]. It enrolled 15,000 patients who were educated regarding kidney function, kidney failure, and renal replacement therapies. The patients chose the dialysis modality after completion of the program, and 55% chose hemodialysis, while 45% chose peritoneal dialysis. Along with predialysis education, effective patient PD training and retraining is important for a successful PD program. A Training program with a well-structured curriculum may be associated with improved outcomes [63]. PD training done at the patient's home has been shown to lower peritonitis rates [64]. Thus patient education and training/retraining is a key target for maintaining patients on PD.

#### 3.2.3. Physician Education and Training

To offset the concerns about nephrologists not being comfortable with PD, training programs must provide fellows adequate exposure to PD. Programs with limited access can offer their trainees elective rotations in centers with larger PD population and have a core curriculum for PD including text and visual aids. For example, the current RRC requirement of minimum 12 months of clinical nephrology can perhaps be increased to 15 or 18 months to get greater PD exposure.

#### 3.2.4. Financial Considerations

The issues related to physician and center reimbursement are more difficult to overcome, but the introduction of bundling of services (treatments, labs, and medications) of dialysis care into one payment will help indirectly by having more patients treated with PD. PD, where there is far less use of injectables as well as utilization of labor, may come out ahead of HD financially and result in increased PD population.

### 3.3. Patient Related

#### 3.3.1. Preventing Patient Burnouts

Patient “Burnout” should be handled with counseling. Providing psychosocial support in the form of home visits by nurses or health aides can minimize this problem. Programs have utilized “assisted peritoneal dialysis”—where the therapy is performed with the assistance of either a visiting nurse or a family member with good results especially in elderly and unplanned starts [65]. Family support has been associated with an increase in peritoneal dialysis eligibility from 63% to 80%, and an increase in peritoneal dialysis utilization from 23% to 39% among patients who had a barrier to self-care peritoneal dialysis [66]. Assisted PD can help certain patients stay on PD longer.

## 4. Conclusion

Peritoneal dialysis utilization continues to be low in many countries. In the USA the recent bundling of services for dialysis care into one payment offers PD as a cost-effective therapy and has generated a renewed interest in the dialysis community which can lead to improved provider expertise and comfort and eventually greater PD utilization. Patient drop-out contributes to the underutilization and is multifactorial, that is, modality related, system related, and patient related. Towards this end, both patient and physician education and comfort with using this modality are critical. Techniques to prevent and minimize episodes of peritonitis, use of more biocompatible solutions in preserving the peritoneal membrane, and careful management of volume status can sustain the patient longer on PD. Use of drugs such as ACE inhibitors and ARBs can preserve the membrane longer. Appropriate and timely radiological and surgical interventions can prevent the malfunction and loss of PD catheters. Psychological help as well as assisted PD with home aide can minimize the phenomenon of “burnouts”.

## Figures and Tables

**Table 1:** Potential causes of transfer to HD.

Modality related
Peritonitis
Tunnel infection, exit site infections
Inadequate dialysis
Ultrafiltration failure
Catheter malfunction

System related

Lack of patient education/training
Transfer to a facility where PD is unavailable
Center effect
Provider expertise
Ownership of dialysis facility

Patient related

Patient fatigue/burnout
Social reasons, family, occupation, and so forth
Geography: distance to travel
Loss of RRF
Malnutrition and/or excess protein loss
Abdominal surgeries/hernia
Stroke or severe illness, limiting the manual dexterity

**Table 2:** Strategies to prevent PD drop-out.

Modality related
Peritonitis prophylaxis and treatment
Membrane preservation: Use of Glucose polymers/ACE inhibitors
Adjust dialysis prescription according to RRF
Correction of catheter malfunction

System related

Better infrastructure to support PD
Patient education/training
Physician and nursing education
Larger PD centers

Patient related

Social support
Psychological counseling (as needed)
Assisted PD

## References

[1] USRDS data system (2009). *USRDS Annual Data Report*.

[2] Ledebo I, Ronco C (2008). The best dialysis therapy? Results from an international survey among nephrology professionals. *NDT Plus*.

[3] Afolalu B, Troidle L, Osayimwen O, Bhargava J, Kitsen J, Finkelstein FO (2009). Technique failure and center size in large cohort of peritoneal dialysis patients in a defined geographic area. *Peritoneal Dialysis International*.

[4] Guo A, Mujais S (2003). Patient and technique survival on peritoneal dialysis in the United States: evaluation in large incident cohorts. *Kidney International*.

[5] Kolesnyk I, Dekker FW, Boeschoten EW, Krediet RT (2010). Time-dependent reasons for peritoneal dialysis technique failure and mortality. *Peritoneal Dialysis International*.

[6] Strippoli GFM, Tong A, Johnson D, Schena FP, Craig JC (2004). Catheter-related interventions to prevent peritonitis in peritoneal dialysis: a systematic review of randomized, controlled trials. *Journal of the American Society of Nephrology*.

[7] Schaubel DE, Blake PG, Fenton SSA (2001). Trends in CAPD technique failure: Canada, 1981–1997. *Peritoneal Dialysis International*.

[8] Jaar BG, Plantinga LC, Crews DC (2009). Timing, causes, predictors and prognosis of switching from peritoneal dialysis to hemodialysis: a prospective study. *BMC Nephrology*.

[9] Daly CD, Campbell MK, MacLeod AM (2001). Do the Y-set and double-bag systems reduce the incidence of CAPD peritonitis?. *Nephrology Dialysis Transplantation*.

[10] Davenport A (2009). Peritonitis remains the major clinical complication of peritoneal dialysis. The London, UK, peritonitis audit 2002-2003. *Peritoneal Dialysis International*.

[11] Twardowski ZJ, Prowant BF, Nichols WK, Nolph KD, Khanna R (1998). Six-year experience with swan neck presternal peritoneal dialysis catheter. *Peritoneal Dialysis International*.

[12] Mujais S, Nolph K, Gokal R (2000). Evaluation and management of ultrafiltration problems in peritoneal dialysis. International Society for Peritoneal Dialysis Ad Hoc Committee on Ultrafiltration Management in Peritoneal Dialysis. *Peritoneal Dialysis International*.

[13] Margetts PJ, Churchill DN (2002). Acquired ultrafiltration dysfunction in peritoneal dialysis patients. *Journal of the American Society of Nephrology*.

[14] Jager KJ, Merkus MP, Dekker FW (1999). Mortality and technique failure in patients starting chronic peritoneal dialysis. Results of the Netherlands Cooperative Study on the Adequacy of Dialysis. *Kidney International*.

[15] Kawaguchi Y, Hasegawa T, Nakayama M, Kubo H, Shigematu T (1997). Issues affecting the longevity of the continuous peritoneal dialysis therapy. *Kidney International, Supplement*.

[16] Mehrotra R, Chiu Y-W, Kalantar-Zadeh K, Bargman J, Vonesh E (2011). Similar outcomes with hemodialysis and peritoneal dialysis in patients with end-stage renal disease. *Archives of Internal Medicine*.

[17] Mehrotra R, Chiu YW, Kalantar-Zadeh K, Vonesh E (2009). The outcomes of continuous ambulatory and automated peritoneal dialysis are similar. *Kidney International*.

[18] Crabtree JH (2006). Rescue and salvage procedures for mechanical and infectious complications of peritoneal dialysis. *International Journal of Artificial Organs*.

[19] Blake PG (1999). Factors affecting international utilization of peritoneal dialysis: implications for increasing utilization in the United States. *Seminars in Dialysis*.

[20] Blake P (2009). Proliferation of hemodialysis units and declining peritoneal dialysis use: an international trend. *American Journal of Kidney Diseases*.

[21] Lameire N, Van Biesen W (2010). Epidemiology of peritoneal dialysis: a story of believers and nonbelievers. *Nature Reviews Nephrology*.

[22] Li PKT, Szeto CC (2008). Success of the peritoneal dialysis programme in Hong Kong. *Nephrology Dialysis Transplantation*.

[23] Li PKT, Chow KM (2005). The clinical and epidemiological aspects of vascular mortality in chronic peritoneal dialysis patients. *Peritoneal Dialysis International*.

[24] Mehrotra R, Khawar O, Duong U (2009). Ownership patterns of dialysis units and peritoneal dialysis in the United States: utilization and outcomes. *American Journal of Kidney Diseases*.

[25] Huisman RM, Nieuwenhuizen MGM, De Charro FT (2002). Patient-related and centre-related factors influencing technique survival of peritoneal dialysis in The Netherlands. *Nephrology Dialysis Transplantation*.

[26] Schaubel DE, Blake PG, Fenton SSA (2001). Effect of renal center characteristics on mortality and technique failure on peritoneal dialysis. *Kidney International*.

[27] Li PKT, Chow KM (2009). Peritoneal dialysis patient selection: characteristics for success. *Advances in Chronic Kidney Disease*.

[28] Plantinga LC, Fink NE, Finkelstein FO, Powe NR, Jaar BG (2009). Association of peritoneal dialysis clinic size with clinical outcomes. *Peritoneal Dialysis International*.

[29] Linas S (2002). Dialysis training in the United States. *American Journal of Kidney Diseases*.

[30] Mehrotra R, Marsh D, Vonesh E, Peters V, Nissenson A (2005). Patient education and access of ESRD patients to renal replacement therapies beyond in-center hemodialysis. *Kidney International*.

[31] Tonelli M, Hemmelgarn B, Culleton B (2007). Mortality of Canadians treated by peritoneal dialysis in remote locations. *Kidney International*.

[32] Piraino B, Perlmutter JA, Holley JL, Bernardini J (1993). Staphylococcus aureus peritonitis is associated with Staphylococcus aureus nasal carriage in peritoneal dialysis patients. *Peritoneal Dialysis International*.

[33] Blowey DL, Warady BA, McFarland KS (1994). The treatment of Staphylococcus aureus nasal carriage in pediatric peritoneal dialysis patients. *Advances in Peritoneal Dialysis*.

[34] Zimmerman SW, Ahrens E, Johnson CA (1991). Randomized controlled trial of prophylactic rifampin for peritoneal dialysis-related infections. *American Journal of Kidney Diseases*.

[35] Bernardini J, Bender F, Florio T (2005). Randomized, double-blind trial of antibiotic exit site cream for prevention of exit site infection in peritoneal dialysis patients. *Journal of the American Society of Nephrology*.

[36] Sanchez AR, Madonia C, Rascon-Pacheco RA (2008). Improved patient/technique survival and peritonitis rates in patients treated with automated peritoneal dialysis when compared to continuous ambulatory peritoneal dialysis in a Mexican PD center. *Kidney International*.

[37] Montenegro J, Saracho R, Gallardo I, Martñnez I, Muñoz R, Quintanilla N (2007). Use of pure bicarbonate-buffered peritoneal dialysis fluid reduces the incidence of CAPD peritonitis. *Nephrology Dialysis Transplantation*.

[38] Ahmad S, Sehmi JS, Ahmad-Zakhi KH, Clemenger M, Levy JB, Brown EA (2006). Impact of new dialysis solutions on peritonitis rates. *Kidney International*.

[39] Szeto CC, Chow KM, Lam CWK (2007). Clinical biocompatibility of a neutral peritoneal dialysis solution with minimal glucose-degradation products—a 1-year randomized control trial. *Nephrology Dialysis Transplantation*.

[40] Williams JD, Topley N, Craig KJ (2004). The Euro-Balance Trial: the effect of a new biocompatible peritoneal dialysis fluid (balance) on the peritoneal membrane. *Kidney International*.

[41] Witowski J, Jörres A, Korybalska K (2003). Glucose degradation products in peritoneal dialysis fluids: do they harm?. *Kidney International*.

[42] Chaudhary K, Khanna R (2010). Biocompatible peritoneal dialysis solutions: do we have one?. *Clinical Journal of the American Society of Nephrology*.

[43] Kuriyama R, Tranaeus A, Ikegami T (2006). Icodextrin reduces mortality and the drop-out rate in Japanese peritoneal dialysis patients. *Advances in Peritoneal Dialysis*.

[44] Davies SJ, Woodrow G, Donovan K (2003). Icodextrin improves the fluid status of peritoneal dialysis patients: results of a double-blind randomized controlled trial. *Journal of the American Society of Nephrology*.

[45] Liao CT, Chen YM, Shiao CC (2009). Rate of decline of residual renal function is associated with all-cause mortality and technique failure in patients on long-term peritoneal dialysis. *Nephrology Dialysis Transplantation*.

[46] Fan SLS, Pile T, Punzalan S, Raftery MJ, Yaqoob MM (2008). Randomized controlled study of biocompatible peritoneal dialysis solutions: effect on residual renal function. *Kidney International*.

[47] Davies SJ (2009). Preserving residual renal function in peritoneal dialysis: volume or biocompatibility. *Nephrology Dialysis Transplantation*.

[48] Moist LM, Port FK, Orzol SM (2000). Predictors of loss of residual renal function among new dialysis patients. *Journal of the American Society of Nephrology*.

[49] Lang SM, Bergner A, Töpfer M, Schiffl H (2001). Preservation of residual renal function in dialysis patients: effects of dialysis-technique-related factors. *Peritoneal Dialysis International*.

[50] Kim DJ, Park JA, Huh W, Kim YG, Oh HY (2001). The effect of hemodialysis during break-in period on residual renal function in CAPD patients. *Peritoneal Dialysis International*.

[51] Suzuki H, Kanno Y, Sugahara S, Okada H, Nakamoto H (2004). Effects of an angiotensin II receptor blocker, valsartan, on residual renal function in patients on CAPD. *American Journal of Kidney Diseases*.

[52] Li PKT, Chow KM, Wong TYH, Leung CB, Szeto CC (2003). Effects of an angiotensin-converting enzyme inhibitor on residual renal function in patients receiving peritoneal dialysis. A randomized, controlled study. *Annals of Internal Medicine*.

[53] Kolesnyk I, Noordzij M, Dekker FW, Boeschoten EW, Krediet RT (2011). Treatment with Angiotensin ii inhibitors and residual renal function in peritoneal dialysis patients. *Peritoneal Dialysis International*.

[54] Kolesnyk I, Noordzij M, Dekker FW, Boeschoten EW, Krediet RT (2009). A positive effect of AII inhibitors on peritoneal membrane function in long-term PD patients. *Nephrology Dialysis Transplantation*.

[55] Freida P, Issad B, Dratwa M (2009). A combined crystalloid and colloid PD solution as a glucose-sparing strategy for volume control in high-transport Apd Patients: a prospective multicenter study. *Peritoneal Dialysis International*.

[56] Davies S, Carlsson O, Simonsen O (2009). The effects of low-sodium peritoneal dialysis fluids on blood pressure, thirst and volume status. *Nephrology Dialysis Transplantation*.

[57] Crabtree JH, Burchette RJ (2009). Effective use of laparoscopy for long-term peritoneal dialysis access. *American Journal of Surgery*.

[58] Jiang Z, Yu X (2011). Advancing the use and quality of peritoneal dialysis by developing a peritoneal dialysis satellite center program. *Peritoneal Dialysis International*.

[59] Lo WK, Kwan TH, Ho YW (2008). Preparing patients for peritoneal dialysis. *Peritoneal Dialysis International*.

[60] Lo WK, Li FK, Choy CBY (2001). A retrospective survey of attitudes toward acceptance of peritoneal dialysis in Chinese end-stage renal failure patients in Hong Kong - From a cultural point of review. *Peritoneal Dialysis International*.

[61] Little J, Irwin A, Marshall T, Rayner H, Smith S (2001). Predicting a patient’s choice of dialysis modality: experience in a United Kingdom renal department. *American Journal of Kidney Diseases*.

[62] Golper T (2001). Patient education: can it maximise the success of therapy?. *Nephrology Dialysis Transplantation*.

[63] Hall G, Bogan A, Dreis S (2004). New directions in peritoneal dialysis patient training. *Nephrology Nursing Journal*.

[64] Castro MJ, Celadilla O, Muñoz I (2002). Home training experience in peritoneal dialysis patients. *EDTNA-ERCA Journal*.

[65] Povlsen JV (2009). Unplanned start on assisted peritoneal dialysis. *Contributions to Nephrology*.

[66] Oliver MJ, Garg AX, Blake PG (2010). Impact of contraindications, barriers to self-care and support on incident peritoneal dialysis utilization. *Nephrology Dialysis Transplantation*.

